# On the Local Treatment of the Diseases of the Air Passages

**Published:** 1855-07

**Authors:** Wm. G. Meacham

**Affiliations:** Warsaw, Wyoming Co., N. Y.


ART. II. — *On the Local Treatment of the Diseases of the Air Passages.* By WM. G. MEACHAM, M. D., Warsaw, Wyoming Co., N. Y.

WARSAW, Wyoming Co., N. Y., April 23, 1855.

MR. EDITOR: — The bold assays, the eagle flights, the Herculian achievements of this famous nineteenth century have, indeed, often and justly been the subject of our Platos' meditations, the burden of our Ciceros' impassioned outbursts, the theme of our Homers' songs; but, may I not ask, have not numberless pretensions to literary, scientific, and artistic excellence been made since its orient dawn, as "baseless as the fabric of a vision?" Are not daily witnessed by us the promulgation of specious theories, the deduction of unfounded inferences, the presentation of misapprehended or distorted facts? Has not mortality as well as truth been belabored in men's demoniac efforts to grasp the bubble fame? Has not "the nineteenth" whereof to mourn as well as boast? Has not "the nineteenth" acquired its *prœnomen* "famous" as well from shameless imposition as from honorable achievement? If it be (and who gainsays?) that "all is not gold that glitters" in this illustrious age, it behooves us thoroughly to test in the crucible of candid, enlightened investigation, every sentiment and theory presented to the public view, and to nothing does this truth apply with greater force than to our own most noble science of medicine, in which all new *truths* are productive of incalculable benefit, but all new *errors* fraught with an immensity of evil to confiding, suffering humanity.

With this great fact deeply imprinted on my mind, I have resolved (perhaps presumptuously, yet not invidiously) on subjecting to various tests a recently-introduced practice of a distinguished New York Professor, one claimed to be tenable not only *in theoria* but also *in experientia*, I mean, the application with the probang of the nitrate of silver in solution to the air-passages, for the relief and cure of various diseases of those important parts.

In conducting this examination I propose to be actuated by no other feeling than an impartial desire for truth, and I trust that I shall in no instance swerve from that straightforward, honorable course in which every searcher for knowledge should scrupulously advance.

This practice is assuredly a "glittering" one, and let me now endeavor to discover whether it is also "gold:" 1st. By subjecting it to the *heat* of scientific investigation; and 2dly, by applying to it the *acids* of experience.

The morbid states of the air-passages in the treatment of which the distinguished professor ascribes efficacy to his favorite remedy, are, as I understand, those the counterparts of which in the more accessible regions of the body have often measureably succumbed under the administration of the same medicinal agent, viz: inflammatory and ulcerous conditions. Nitrate of silver, doubtless, says the Prof., has been found of service in subacute and chronic conjunctivitis: why may it not be equally efficacious in similar varieties of bronchitis? Nitrate of silver is one of the puissant weapons wielded against various forms of external ulceration; why may it not strike as effective blows against pulmonary vomicæ?

In the abstract these are certainly rational queries; but, perchance, a little attention to some anatomical, physiological and pathological details, will serve to clip these Samsons of some of their power over medical minds. Subacute and chronic mucous inflammation, it is undoubtedly true, are often modified by local applications of the nitrate of silver, but yet it must be remembered that in battling with these diseases this remedial agent is merely an auxiliary force, not the main army—that consists in *general* treatment. Hence, though theoretically this article of the materia medica may lay claim to serviceableness in the management of bronchitis, still it is only a branch, not the root and trunk of its successful treatment.

Now if it can be shown that the risks incurred in the employment of this therapeutical means are sufficiently great to excite no inconsiderable degree of alarm, we are indisputably justified in ostracizing the practice. This I

will assay to do. And, first, let me direct attention to the mechanical effects of the use of the probang.

The introduction of this instrument into the larynx, even though performed by the most skillful and careful operator, cannot be other than a *harsh* procedure, more or less compromising the integrity of the chordœ vocales; indeed, that of the lining membrane of the entire cavity, by nature ordained to be entered by the breath of heaven alone. That this harshness is not a vain imagination, but a grave reality, reason and experience mournfully attest —a harshness which owes no small share of its intensity to the convulsive contractions of the laryngeal muscles induced by the irritation of the foreign substance.

Again. I think we may, without rendering ourselves in the least obnoxious to the charge of superstition, assert that nature's indications are, to a certain extent, to be regarded as well in medical treatment as in other matters. Now, in the case of what other canal of the body do we discern such precaution taken to occlude its passage against every thing save that which is its appropriate element, as in the instance of the larynx? At its entrance, the rima glottidis, is stationed that faithful sentinel, the epiglottis, which freely gives entrance and exit to its old friend, the air; but bravely resists whatever else may present itself, until overpowered by superior strength. Moreover, who has not witnessed the instantaneous spasmodic action, often imperiling existence, occasioned by the accidental admission of a crumb of bread, or some other equally insignificant substance, into the larynx? Who that has seen the probang inserted has not also seen this tumultuous consequence? Do such provisions pertain to the alimentary canal, the urethra, the vagina, the meatus auditorius externus, the Eustachian tube? Nature has, indeed, supplied defences, of some character or other, to all her channels in communication with the exterior, but none which so emphatically interdict the ingress of every thing save the proper, as do those of the larynx. She has, indeed, planted vibrissæ in the anterior nares, and guarded the entrances of the uterus and vesica urinaria by sphincters; but these defences are as inferior in efficacy to those of the air-passages, as is the danger resulting from the admission of foreign materials less.

At first sight this may have the semblance of magnifying molehills into mountains, but I sincerely believe that these visible and audible demonstrations of nature are not to be ruthlessly trampled in the dust as unworthy of notice; and I also believe that every introduction of the probang has a decided tendency, mechanically, to occasion inflammatory excitement when it is absent, and to exasperate it when it exists.

Another untoward event which may possibly attend the use of the probang, as proven in the recent personal experience of the New York Professor, is the escape of the instrument from the operator's hand, and its precipitate descent down the trachea, an accident which at once suggests its great hazard.

Again. The nitrate of silver itself not unfrequently heaps coals, rather than pours water, upon the fire of inflammation. Who that has had any, however little, experience in its topical use, can controvert this statement? Who that has employed it in stomatitis and in conjunctivitis, has not seen their slumbering embers roused into ungovernable fury by its injudicious or untimely use? True, you may say that any remedy may be improperly administered, and that, therefore, to maintain my ground, it becomes necessary for me to denounce all medicinal interference. Not so. Upon external parts, and even upon those internal that are visible, a powerful substance like the one under consideration, can be used with comparative safety, for its effects are evident to the sight, and any error in administration, such as too great strength of solution, too frequent application, or, it may be, its inaptness to the condition and circumstances, can readily be appreciated and rectified, and its evil consequences intercepted. But the cavity of the trachea is a sealed book, a rayless cavern, to the physician. His solutions of too great strength, or his "spongings" of too frequent repetition, may do injury which may not manifest itself through its constitutional effects until long after it has reached that pitch from which it will be in vain to attempt to repress it—until long after it has attained that severity against which the best directed efforts will be entirely useless.

Once more. The solution must, by its own weight, descend along the air-tubes until, at last, what is not arrested by the mucous membrane will be accumulated in the pulmonary vesicles, and there remain until absorption and evaporation have caused its removal. Hence it appears that the force of the remedy will be expended upon the mucous membrane of the air-cells. Now in what varieties of disease is this the indication? I leave the intelligent physician to answer this query.

But again. The long continuance of the solution in the cells must inevitably defeat the very object desired, for who does not know that a protracted application of this agent will occasion the very condition for the relief of which it is prescribed. In fact, if of adequate strength and in requisite quantity, it may even cauterize the delicate lining of the vescicles, and, still remaining, then attack the pulmonary parenchyma, equally delicate, and perchance cease not its ravages until it has effected a passage into the pleural sac. That this is not impossible, nor yet improbable, is the legitimate inference deducible from its known properties and effects. The evil, if not fatal, consequences of such an issue, among which are pneumonitis, pulmonary emphysema, pleuritis, pneumothorax, &c., I scarcely need mention, since they will readily be suggested to every medical mind. With this formidable array of not merely possible, but highly probable results of its rise before us, does it not become us to hesitate at its recommendation and adoption?

Further. As I before remarked, the nitrate of silver treatment in mucous inflammation, is at least (to say nothing of the many and great risks attendant upon its employment) of but minor importance. Who assays to treat a urethritis with topical use of this chemical alone? Or who puts his chief reliance upon it? Who is not convinced, and who does not act upon that conviction, that his hopes rest upon general and local depletion, counterirritation, &c.; or it may chance be, upon roborant measures? Now shall we subject our bronchitic patient to all the risks of casualty inseparable from the probang and its escharotic freight to attempt to secure—but a secondary benefit?

Again. Although in idiopathic bronchitis but little gain is to be expected from this agent, in the symptomatic or secondary varieties (and these constitute the great bulk of cases) infinitely less is to be hoped for in its administration. Indeed, in the use of the nitrate in these latter cases we boldly oppose ourselves to the fundamental doctrine, never to prescribe for symptoms. Take, for example, a case of bronchitis consequent on phthisis pulmonalis—a very common, perhaps an invariable sequence—what possible radical good can reasonably be expected from regarding the former so long as the latter, its cause, continues in activity? 'T is like directing the vehemence of artillery against some unimportant appendage rather than upon the fortress itself. 'T is like attempting to arrest a fountain's flow by damming up one of the many streams that issue therefrom. The Prof.'s favorite practice in such a case, can, under the most favorable circumstances, but relieve the bronchitis temporarily, for as long as its excitant, phthisis, exists, so long will it continually recur. But, as has already been observed, a temporarily favorable issue is far from being at all times confidently anticipated. In this condition of things is it expedient to incur the formidable hazards just alluded to in the uncertain hope of securing a transient secondary advantage? Reason and the acknowledged principles of medical science unhesitatingly answer, No.

Now a word in reference to the application of this therapeutic agent to the consequences of inflammation of the mucous lining of the air-passages, effusion of serum, hæmorrhage, induration, hypertrophy, suppuration, ulceration, and gangrene. Applicability to but one of these effects, ulceration does any authority or experience, so far as I am informed, ascribe to the nitrate of silver, while on each of all the others its influence is decidedly prejudicial. Moreover, it is well known that to secure its beneficial action in external ulceration it is generally found necessary to employ solutions of very considerable strength, often the solid stick, while observation has taught that the ulcerated space should alone be touched, or at most, but an extremely limited portion of the surrounding parts.

Now, let me inquire, can we at all times, or even generally, unmistakably decide which of the preceding list of consequences is the one for which we may have been called on to prescribe? Were the surface of the body, the mouth, the fauces, the vagina or any other visible portion, the seat of the difficulty, our diagnosis would be sufficiently easy, but, unquestionably, when the air-passages are the locality the perplexity is greatly enhanced, if it be not insurmountable. Are we, then, justified in the use of this remedy in this dark obscurity of diagnosis?

How is it possible, moreover, to ascertain the precise situation of the ulcerated portion of the air-psssages, even though we suceeed in substantiating the truth of the existence of ulceration, or, in the improbable event of accomplishing even this, how are we to proceed to limit our application to the ulcers, in conformity to the rule of authorized practice? What serious harm may not the powerful solutions requisite to affect in a sanative manner the ulcerated parts, entail on those intact? As before remarked, what serious injury may they not inflict upon the delicate lung-structure, scarcely less delicate than gossamer?

Last. Let me direct attention to that as yet invincible foe of the human race, pulmonary consumption, for the relief of which the remedy under consideration, topically employed, has been declared by some to possess efficacy. The stage of this disease in which this agent is asserted to have proved serviceable, is that in which there exist in the lungs cavities so communicating with the bronchi and bronchæ as to admit of the passage of the remedy in solution into them. Now, as in bronchitis superinduced by phthisis, so also in ulceration of the pulmonary structure attendant upon that fearful scourge, all local medication is but putting new wine into old bottles, new cloth into old garments. How can we justly anticipate, even from the best-directed topical treatment, any but an ephemeral arrest of the fatally-destructive process, so long as tuberculous material still remains to irritate and inflame the circumjacent parts? We may, by a bare possibility, succeed in transiently repressing the flame to a limited extent, only to witness it burst forth with renewed vigor, or to rage more furiously elsewhere. How shall we be enabled, moreover, unmistakably to introduce the medicament into the cavity? Can we at all times, or even generally, aye, *ever* avoid the passage of more or less of the solution (which in this case should be of unusual strength) into lobules and lobes unaffected with ulceration, where its presence would act as the match to the train! To attempt to check this pulmonary ulceration with the feeble restraints of the nitrate of silver, is but binding our strong enemy with *threads;* while the unavoidable passage of the potent chemical into parts intact, is entwining *cords* about our faithful and effective ally. Add to this the injurious results of the probang's use, as referred to above, and I must confess I see but little to encourage the physician to anticipate much save untoward issue in pulmonary ulceration from the treatment under consideration.

Having now submitted this therapeutical plan to the first of the proposed tests, that by the *heat* of scientific investigation, and found it a substance possessing neither, to my eye, the lustre, in my balance, the weight, beneath my mind's "roller," the malleability, nor in my intellective experiments, the conducting power, of gold; let me, in the next place, apply to it the test of the *acids* of experience. And this let me proceed briefly to do by referring to the practice of the worthy N. Y. Prof. and, indeed, of all who have resorted to the tracheal probang. What do their experience and their records show? An increased number of recoveries? A prolongation of life? An amelioration of symptoms? I unhesitatingly answer, No. The monsters rage yet more, more furious from their slight restraint. The acids of experience, each and all, corrode this theory, dissolve this practice. The *nitric acid* of "favorable cases," no less than the *aqua regia* of "hopeless cases," consumes this "glittering" but not "golden" treatment.

Doubtless, were its accompanying and succeeding inconveniencies and dangers withdrawn, the nitrate of silver might be productive of an equal amount of good to the mucous linings of the larynx, the trachea, and the larger bronchi as is seen to attend its use in *simple* conjunctivitis, vaginitis, and urethritis, but not in the *specific* varieties of these diseases, for upon them this remedy unquestionably acts with an energy and efficacy bordering on "specific" virtue. But I humbly, yet confidently, aver that it were far better that not a drop of this solution should ever come in contact with the mucous lining of the bronchiæ and pulmonary air-cells!

The distinguished Prof., whose name is inseparably bound up in this system of therapeutics, or some other plodding disciple of Galen, may yet succeed, by the force of his inventive genius, (and I trust he may,) in devising some mechanical appliance, free from the weighty objections to the probang, by which he may be enabled to apply a solution of any required strength to any given locality, however limited or spacious in extent. Should our means of diagnosis, moreover, be at any future time so increased and perfected as to remove all doubt in relation to the nature and the seat of the malady, then we might prescribe this agent in idiopathic bronchitis with the assurance of *assisting in*, but not *accomplishing* a restoration to health.

In the hope that this system may, if it be possible, be placed upon a firm foundation of rational, scientific and experimental consistency, and wishing its great champion success as great as his perseverance and assiduity deserve,

I subscribe myself,

Your fellow Knight-of-the-Lancet,

WM. G. MEACHAM.

